# Supplementary material for: FOXO Dictates Initiation of B Cell Development and Myeloid Restriction in Common Lymphoid Progenitors
Source: Front Immunol. 2022 May 4;13:880668. doi: 10.3389/fimmu.2022.880668 (PMC9116193; doi:10.3389/fimmu.2022.880668)
Supplement: Supplementary file 1 [file DataSheet_1.zip › data sheet 1/Figure S1-5.pdf]

Fig. S1

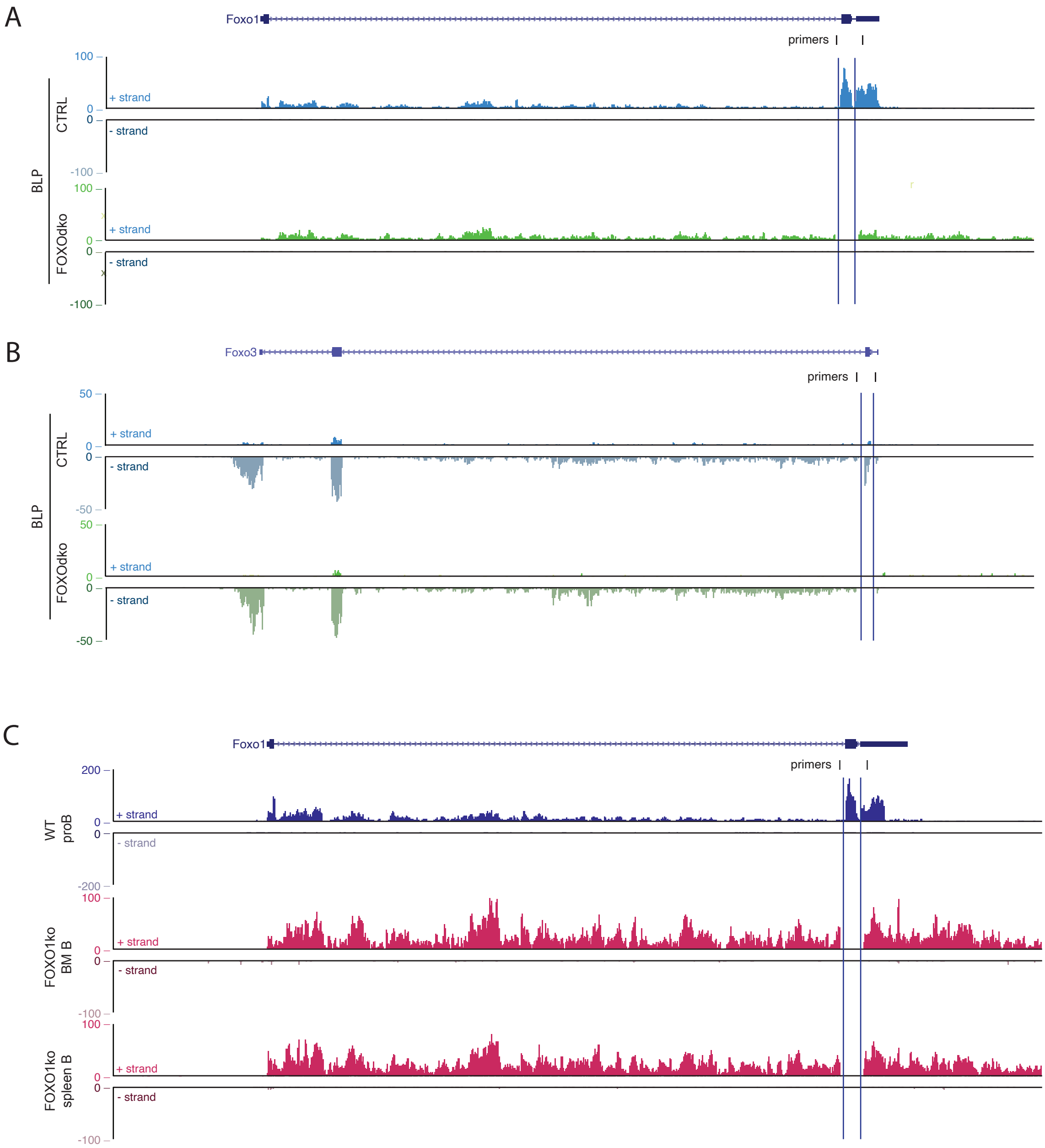
A
Foxo1
primers
100
0
0
-100
+ strand
- strand
CTRL
FOXOdko
BLP
B
Foxo3
primers
50
0
0
-50
+ strand
- strand
CTRL
FOXOdko
BLP
C
Foxo1
primers
200
0
0
-200
100
0
0
-100
+ strand
- strand
WT proB
FOXO1ko BM B
FOXO1ko spleen B

Figure S1. The conditional Foxo alleles are efficiently deleted by Vav-iCre. (A-C) Genome browser tracks showing the lack of the floxed *Foxo1* and/or *Foxo3* exons in transcripts from FOXOdko (*Foxo1*^{ff}*Foxo3*^{ff}*VAV*^{iCre}) BLPs (A-B) and FOXO1ko B cells (C). Axis display read counts (normalized to 10 million total reads) on the plus and minus strand respectively. The position of primers flanking the floxed exons are indicated.

Fig. S2

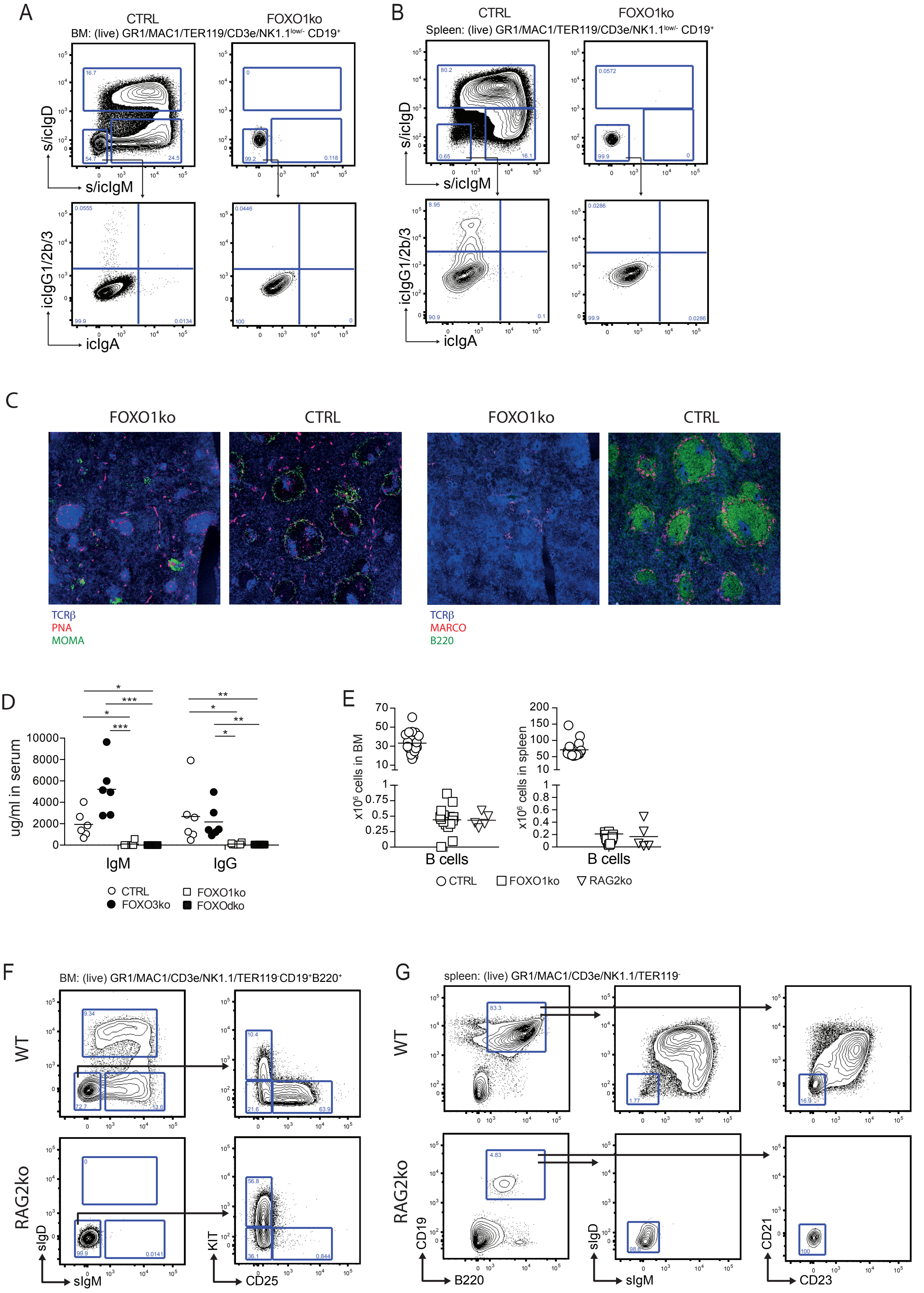
A
CTRL
FOXO1ko
BM: (live) GR1/MAC1/TER119/CD3e/NK1.1low/- CD19+
s/icIgD
s/icIgM
icIgG1/2b/3
icIgA
B
CTRL
FOXO1ko
Spleen: (live) GR1/MAC1/TER119/CD3e/NK1.1low/- CD19+
C
FOXO1ko
CTRL
TCRβ
PNA
MOMA
MARCO
B220
D
ug/ml in serum
IgM
IgG
CTRL
FOXO3ko
FOXO1ko
FOXOdko
E
x10^6 cells in BM
x10^6 cells in spleen
B cells
CTRL
FOXO1ko
RAG2ko
F
BM: (live) GR1/MAC1/CD3e/NK1.1/TER119-CD19+B220+
WT
RAG2ko
sIgD
sIgM
KIT
CD25
G
spleen: (live) GR1/MAC1/CD3e/NK1.1/TER119-
CD19
B220
CD21
CD23

Figure S2. Characterization of residual B cells in FOXO and RAG knockout mice. (A-B) Total number B cells in (A) BM and (B) spleen. In panel A-B and H-I: each dot represents data from an individual mouse; p-values were calculated using the Kruskal Wallis test with Dunn's test of multiple comparisons; *, ** and *** indicating p-values <0.05, <0.01 and <0.001 respectively. (C-D) Gating strategy for identification of B cells in (C) BM and (D) spleen. (E-F) Representative intracellular (ic) and surface (s) staining of IgA, D, G and M on (E) BM and (F) spleen B cells. (G) Immunohistochemistry on spleen sections. Representative areas are shown. (H) ELISA detection of IgM and IgG in serum. (I) Total number of B cells in the indicated organ. (J) Gating strategy for identification of BM B lineage cells. (K) Gating strategy for identification of spleen B cell subsets.

Fig. S3

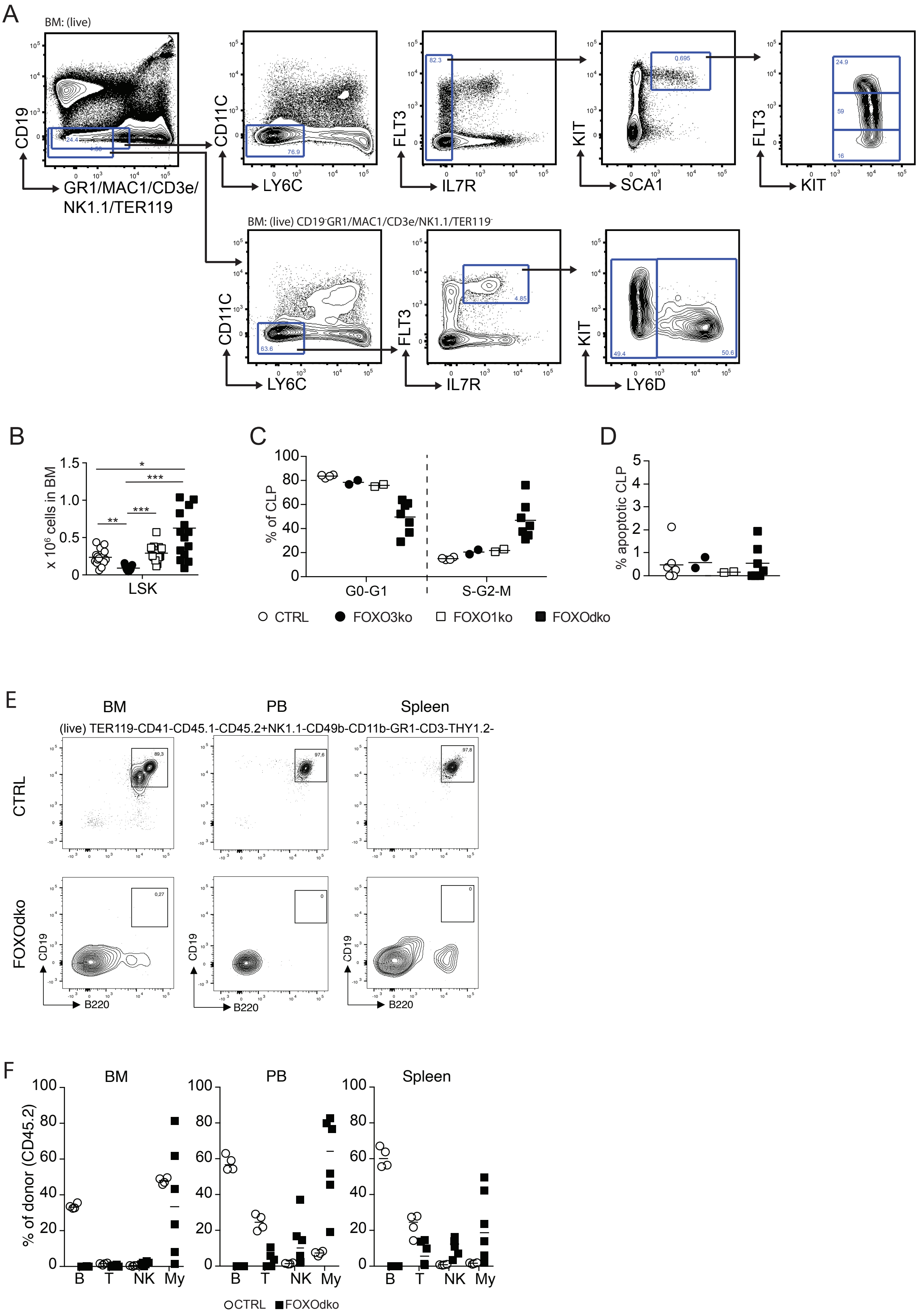
A
BM: (live)
CD19
GR1/MAC1/CD3e/NK1.1/TER119
CD11C
LY6C
FLT3
IL7R
KIT
SCA1
KIT
BM: (live) CD19-GR1/MAC1/CD3e/NK1.1/TER119-
CD11C
LY6C
FLT3
IL7R
KIT
LY6D
B
x 10^6 cells in BM
LSK
C
% of CLP
G0-G1
S-G2-M
D
% apoptotic CLP
CTRL
FOXO3ko
FOXO1ko
FOXOdko
E
BM
PB
Spleen
(live) TER119-CD41-CD45.1-CD45.2+NK1.1-CD49b-CD11b-GR1-CD3-THY1.2-
CTRL
FOXOdko
CD19
B220
F
BM
PB
Spleen
% of donor (CD45.2)
B
T
NK
My
CTRL
FOXOdko

Figure S3. Characterization of number, cycling, apoptosis and reconstitution of FOXO deficient progenitors. (A) Gating strategy for identification of stem and progenitor cells. (B) total number of LSK cells in BM. In panels B-D: each dot represents data from an individual mouse. In panel B: p-values were calculated using the Kruskal Wallis test with Dunn's test of multiple comparisons; *, ** and *** indicating p-values <0.05, <0.01 and <0.001 respectively. (C) Percentage of CLPs in the indicated cell cycle stage as determined by Ki67 and DAPI staining. (D) Percentage of apoptotic CLPs as determined by Annexin V staining. (E) Gating strategy for identification of B cells in bone marrow (BM), peripheral blood (PB) and spleen from lethally irradiated CD45.1 mice transplanted with $5x10^6$ (CD45.2) donor (CTRL or FOXOdko) and $0.2x10^6$ support (CD45.1) BM cells. (F) % of the donor ($CD45.2^+$) derived cells constituted the indicated cell-types in BM (left), PB (middle) and spleen (right). Analysis was performed 12 weeks post transplantation and each dot represents data from an individual mouse.

Fig. S4

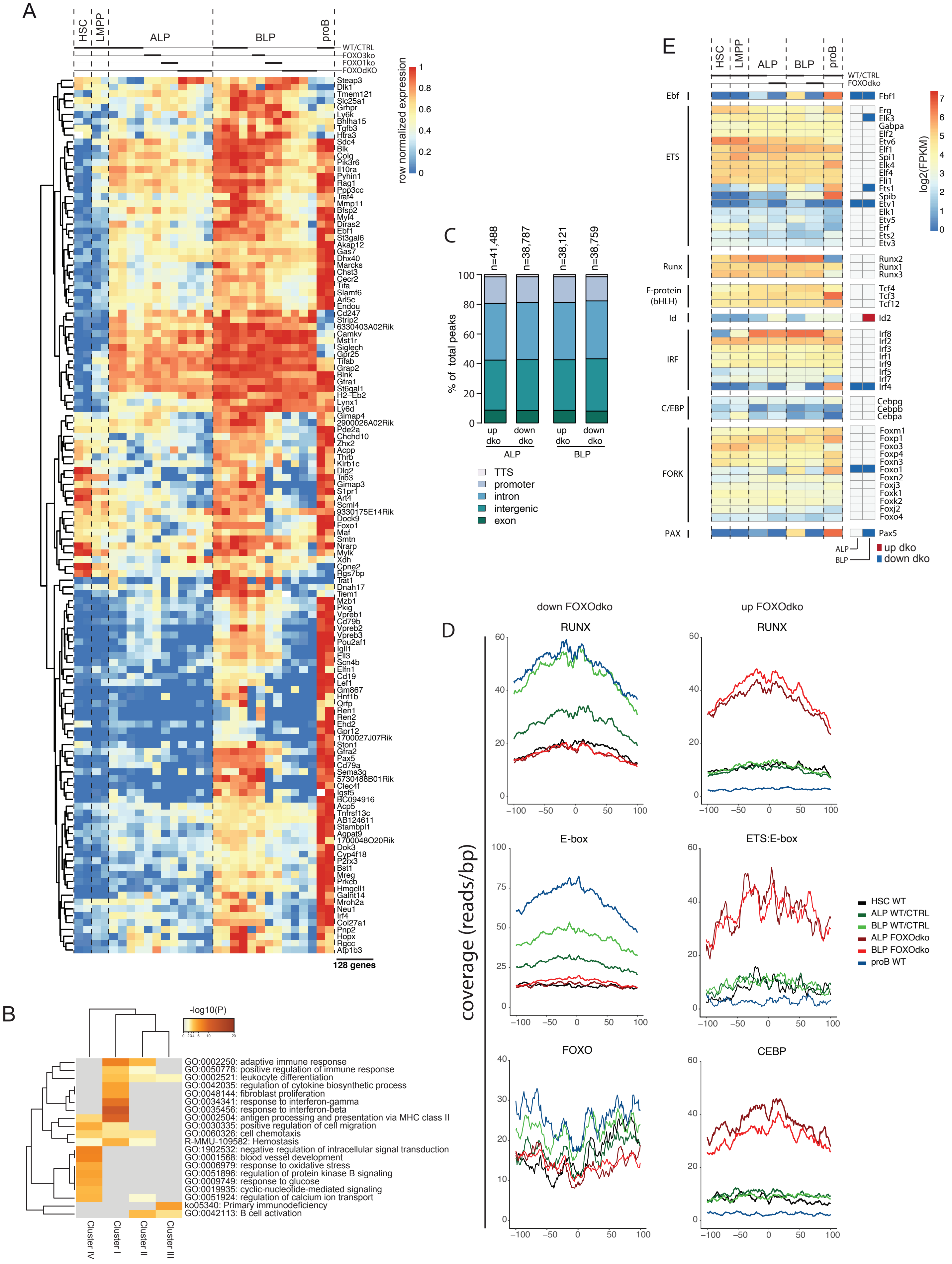
A
HSC
LMPP
ALP
BLP
proB
WT/CTRL
FOXO3ko
FOXO1ko
FOXOdKO
row normalized expression
1
0.8
0.6
0.4
0.2
0
Steap3 Dlk1 Tmem121 Slc25a1 Grhpr Ly6k Bhlha15 Tgfb3 Htra3 Sdc4 Blk Colq Pik3r6 Il10ra Pyhin1 Rag1 Ppp3cc Traf4 Mmp11 Bfsp2 Myl4 Diras2 Ebf1 St3gal6 Akap12 Gas7 Dhx40 Marcks Chst3 Cecr2 Tifa Slamf6 Arl5c Endou Cd247 Strip2 6330403A02Rik Camkv Mst1r Siglech Gpr25 Tifab Grap2 Blnk Gfra1 St6gal1 H2-Eb2 Lynx1 Ly6d Gimap4 2900026A02Rik Pde2a Chchd10 Zhx2 Acpp Thrb Klrb1c Dlg2 Tiib3 Gimap3 S1pr1 Art4 Scml4 9330175E14Rik Dock9 Foxo1 Maf Smtn Nrarp Mylk Xdh Cpne2 Rgs7bp Trat1 Dnah17 Trem1 Mzb1 Pkig Vpreb1 Cd79b Vpreb2 Vpreb3 Pou2af1 Igll1 Ell3 Scn4b Elfn1 Cd19 Lef1 Gm867 Hnf1b Qrfp Ren1 Ren2 Ehd2 Gpr12 1700027J07Rik Ston1 Gfra2 Pax5 Cd79a Sema3g 5730488B01Rik Clec4f Igsf5 BC094916 Acp5 Tnfrsf13c AB124611 Stambpl1 Agpat9 1700048O20Rik Dok3 Cyp4f18 P2rx3 Bst1 Mreg Prkcb Hmgcll1 Galnt14 Mroh2a Neu1 Irf4 Col27a1 Pnp2 Hopx Rgcc Afp1b3
128 genes
B
-log10(P)
GO:0002250: adaptive immune response
GO:0050778: positive regulation of immune response
GO:0002521: leukocyte differentiation
GO:0042035: regulation of cytokine biosynthetic process
GO:0048144: fibroblast proliferation
GO:0034341: response to interferon-gamma
GO:0035456: response to interferon-beta
GO:0002504: antigen processing and presentation via MHC class II
GO:0030335: positive regulation of cell migration
GO:0060326: cell chemotaxis
R-MMU-109582: Hemostasis
GO:1902532: negative regulation of intracellular signal transduction
GO:0001568: blood vessel development
GO:0006979: response to oxidative stress
GO:0051896: regulation of protein kinase B signaling
GO:0009749: response to glucose
GO:0019935: cyclic-nucleotide-mediated signaling
GO:0051924: regulation of calcium ion transport
ko05340: Primary immunodeficiency
GO:0042113: B cell activation
Cluster IV
Cluster I
Cluster II
Cluster III
C
n=41,488
n=38,787
n=38,121
n=38,759
% of total peaks
100
80
60
40
20
0
up dko
down dko
up dko
down dko
ALP
BLP
TTS
promoter
intron
intergenic
exon
D
coverage (reads/bp)
down FOXOdko
up FOXOdko
RUNX
E-box
ETS:E-box
FOXO
CEBP
-100 -50 0 50 100
HSC WT
ALP WT/CTRL
BLP WT/CTRL
ALP FOXOdko
BLP FOXOdko
proB WT
E
HSC
LMPP
ALP
BLP
proB
WT/CTRL
FOXOdko
log2(FPKM)
7
6
5
4
3
2
1
0
Ebf
Ebf1
ETS
Erg Elk3 Gabpa Elf2 Etv6 Elf1 Spi1 Elk4 Elf4 Fli1 Ets1 Spib Etv1 Elk1 Etv5 Erf Ets2 Etv3
Runx
Runx2 Runx1 Runx3
E-protein (bHLH)
Tcf4 Tcf3 Tcf12
Id
Id2
IRF
Irf8 Irf2 Irf3 Irf1 Irf9 Irf5 Irf7 Irf4
C/EBP
Cebpg Cebpb Cebpa
FORK
Foxm1 Foxp1 Foxo3 Foxp4 Foxn3 Foxo1 Foxn2 Foxj3 Foxk1 Foxk2 Foxj2 Foxo4
PAX
Pax5
ALP
BLP
up dko
down dko

Figure S4. Complementary information related to gene expression and chromatin accessibility changes in FOXO deficient CLPs. (A) Hierarchical clustering of genes differentially up-regulated (Bonferroni corrected p-value <0.05 and 2-fold difference and ≥ 30 reads in at least two samples) in the ALP to BLP transition. (B) Gene set enrichment (Metascape) analysis of clusters I-IV from Figure 4D. (C) Bar graph illustrating the percentage of open chromatin regions (ATACseq peaks) within different genomic locations. The total number of peaks identified in each population is indicated above the bar-graphs. (D) Transposase integration-based cut-profiles of DARs with: decreased ATACseq signal containing RUNX, E-protein and FOXO binding sites (left); or increased ATACseq signals containing RUNX, ETS:Ebox and CEBP binding sites (right) in FOXOdko CLPs. (E) Heatmap illustrating expression in log2(FPKM) of expressed TFs (FPKM>1) from the indicated families.

Fig. S5

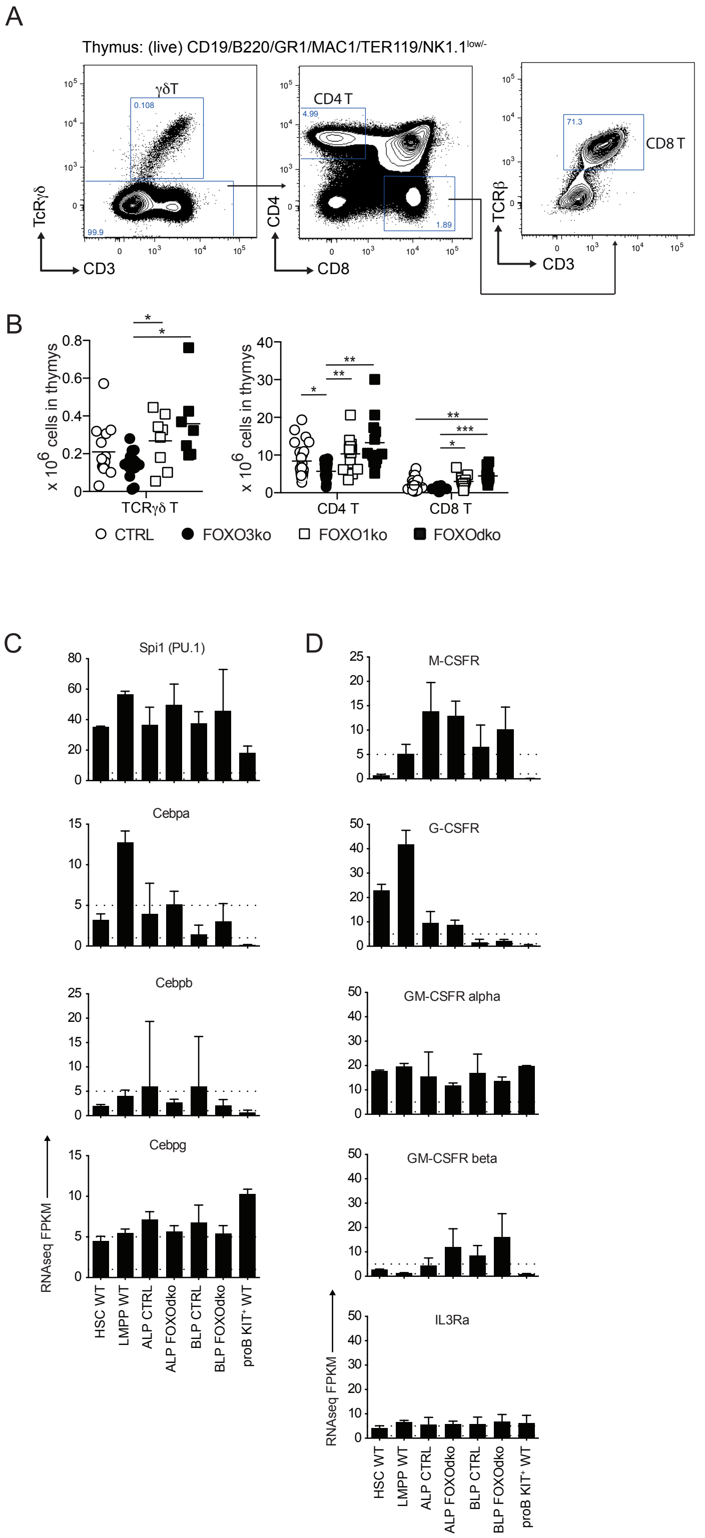
A
Thymus: (live) CD19/B220/GR1/MAC1/TER119/NK1.1low/-
γδT
CD4 T
CD8 T
TcRγδ
CD3
CD4
CD8
TCRβ
CD3
B
x 10^6 cells in thymys
TCRγδ T
CD4 T
CD8 T
CTRL
FOXO3ko
FOXO1ko
FOXOdko
C
D
Spi1 (PU.1)
Cebpa
Cebpb
Cebpg
M-CSFR
G-CSFR
GM-CSFR alpha
GM-CSFR beta
IL3Ra
RNAseq FPKM
HSC WT
LMPP WT
ALP CTRL
ALP FOXOdko
BLP CTRL
BLP FOXOdko
proB KIT+ WT

Figure S5. Characterization of thymic populations and expression of myeloid associated genes. (A) Gating strategy for identification of thymic populations. (B) Total number of indicated T-lineage cells. Each dot represents data from an individual mouse; p-values were calculated using the Kruskal Wallis test with Dunn's test of multiple comparisons; *, ** and *** indicating p-values <0.05, <0.01 and <0.001 respectively. (C-D) Expression (FPKM) of transcription factor (C) and cell surface receptor (D) genes associated with myeloid development.
